# Prenatal alcohol exposure is associated with early motor, but not language development in a South African cohort

**DOI:** 10.1017/neu.2019.51

**Published:** 2020-06

**Authors:** Gaironeesa Hendricks, Susan Malcolm-Smith, Dan J. Stein, Heather J. Zar, Catherine J. Wedderburn, Raymond T. Nhapi, Tawanda Chivese, Colleen M. Adnams, Kirsten A. Donald

**Affiliations:** 1 Department of Psychiatry and Mental Health, and SAMRC Unit on Risk and Resilience, University of Cape Town, Cape Town, South Africa; 2 Department of Paediatrics and Child Health, Red Cross War Memorial Children’s Hospital, University of Cape Town, Cape Town, South Africa; 3 Department of Psychology, Applied Cognitive Science and Experimental Neuropsychology Team, University of Cape Town, Cape Town, South Africa; 4 Department of Psychiatry and Mental Health, University of Cape Town, Cape Town, South Africa; 5 SAMRC Unit on Child and Adolescent Health, University of Cape Town, Cape Town, South Africa; 6 Department of Clinical Research, London School of Hygiene & Tropical Medicine, London, UK; 7 Department of Population Health, College of Medicine, Qatar University, Doha, Qatar; 8 Neuroscience Institute, University of Cape Town, Cape Town, South Africa

**Keywords:** prenatal alcohol exposure, motor development, language development, neurodevelopment

## Abstract

**Objective:**

To investigate the association of prenatal alcohol exposure (PAE) and early neurodevelopment in the first 2 years of life, adjusting for maternal socio-demographic and psychosocial factors, in the Drakenstein Child Health Study (DCHS), a South African birth cohort study.

**Methods:**

The DCHS comprises a population-based birth cohort of 1143 children, of which a subsample completed the Bayley Scales of Infant Development-III (BSID-III) at 6 (*n* = 260) and 24 months of age (*n* = 734). A subset of alcohol-exposed and -unexposed children was included in this analysis at age 6 (*n* = 52 exposed; *n* = 104 unexposed) and 24 months (*n* = 92 exposed; *n* = 184 unexposed). Multiple hierarchical regression was used to explore the associations of PAE with motor and language development.

**Results:**

PAE was significantly associated with decreased gross motor [odds ratio (OR) = 0.16, 95% confidence interval (CI) = 0.06–0.44, *p* = 0.001] or fine motor (OR = 0.16, 95% CI = 0.06–0.46, *p* = 0.001) functioning after adjusting for maternal socio-demographic and psychosocial factors at 6 months of age only. No significant effects were found in either receptive or expressive communication and cognitive outcomes at either time points.

**Conclusion:**

PAE has potentially important consequences for motor development in the first 2 years of life, a period during which the most rapid growth and maturation occur. These findings highlight the importance of identifying high-risk families in order to provide preventive interventions, particularly in antenatal clinics and early intervention services.

## Significant outcomes

- PAE had a significant impact on motor functioning after adjusting for variety of socio-demographic and psychosocial factors at 6 months of age, but not on cognitive or language development.
- The findings of this study highlight the importance of identifying psychosocial risk factors, particularly in antenatal clinics and early intervention services in a South African context.

## Limitations

- The BSID-III tool assesses general ability of a given task but may have low sensitivity for detecting minor developmental impairments especially during infancy.
- Language impairments are very subtle in the early years and may be more difficult to identify impairments than in other domains.
- A small sample size may have reduced the power of the study, and findings may not be generalisable to other populations.

## Introduction

Prenatal alcohol exposure (PAE) has been recognised as a major global public health concern. A recent study estimated 9.8% of mothers consumed alcohol during pregnancy and 4.3% were heavy drinkers (defined as an average of two or more drinks per day; Popova *et al.*, 2017). Estimated global prevalence rates of foetal alcohol spectrum disorders (FASDs) have been reported at 7.7 (4.9–11.7) per 1000 children (Olivier *et al.*, 2016). In low- and middle-income countries (LMICs), such as South Africa, the estimated prevalence of FASDs is as high as 111.1 per 1000 children in some communities (Olivier *et al.*, 2016). The majority of previous studies exploring the impact of PAE on child development in the context of psychobiological and psychosocial factors have been performed in high-income countries, even though higher rates of PAE, poverty, post-traumatic stress disorder (PTSD) and depression exist in LMICs (May *et al.*, 2008; Keen *et al.*, 2010; Flak *et al.*, 2014). The research taking into account contextual factors such as those cited above underscores the importance of examining the adverse effects of PAE in young children (May *et al.*, 2008; Flak *et al.*, 2014), within the broader context of psychosocial and environmental risk factors that may additionally influence not only early neurodevelopmental outcomes but also lifelong health trajectories.

The adverse effects of PAE manifest a continuum of disorders, namely, FASDs. Foetal alcohol syndrome (FAS) is a pattern of often irreversible physical and mental birth deficiencies (Nayak & Murthy, 2008; Safe *et al.*, 2018), while alcohol-related neurodevelopmental disorder (ARND) and alcohol-related birth defects are described as conditions in which the exposed child demonstrates some but not all features of FAS (Sokol *et al.*, 2003). Previous studies have shown that FAS and ARND are associated with a range of impairments in motor functioning, reading comprehension or executive functioning in the early school years (Adnams *et al.*, 2001; Cone-Wesson, 2005; Comasco *et al.*, 2018). Safe *et al.* (2018), for example, have reported that children with FAS displayed both motor function and language impairments at 12 years of age, while Coggins *et al.* (2007) found that school-aged children with FASDs often exhibit clinically meaningful deficits in language and social communication between 6 and 12 years of age. Previous work by Viholainen *et al.* (2006) reported that impaired language development has also been found to be precursor of problems with motor functioning in the school years.

While there is a rapidly growing literature detailing the effects of PAE on neurodevelopmental outcomes in school-going children, comparable data across motor and language functioning are limited in very young children. A previous cross-sectional study assessed specific developmental domains and found PAE deficits at 12 months of age: motor coordination and gross motor functioning (Hutchinson *et al.*, 2019). Other cross-sectional analyses found that FAS was associated with abnormal walking and balance (Kaplan-Estrin *et al.*, 1999; O’Leary, 2004; Kalberg *et al.*, 2006; Henderson *et al.*, 2007; Mattson *et al.*, 2011) and deficits in receptive and expressive communication through 2 years (Kodituwakku, 2007; O’leary *et al.*, 2009; Kodituwakku *et al.*, 2011). However, very few studies included data at different time points in the first 2 years of life (see Hendricks *et al.*, 2018). Of the few studies exploring developmental impairments over time, heavy alcohol exposure was significantly associated with delayed motor functioning in toddlers between 12 and 17 months but not at 24 months of age (Fried & Watkinson, 1988; Jacobson & Jacobson, 2002; Davies *et al.*, 2011). However, the heterogeneity in designs and methodologies of previous studies limits the ability to interpret results across different age cohorts. For example, the impact of maternal alcohol consumption on child outcomes using a clinical diagnosis of FAS without focus on children who do not meet the FASD criteria was reported in only one study (Davies *et al.*, 2011).

Much of the longitudinal research describing the developmental outcomes in early childhood has been conducted in well-resourced settings (Fried & Watkinson, 1990; Fried *et al.*, 1992; Kaplan-Estrin *et al.*, 1999), less is known about the effects of PAE on early neurodevelopmental outcomes at different time points in LMICs and much of the work published to date has lacked control groups and/or has adjusted for very few confounders (maternal age, gestation, birth weight and parity; Fried & Watkinson, 1988, 1990; Fried *et al.*, 1992; Jacobson & Jacobson, 2002). Few studies have adjusted for additional psychosocial factors, such as maternal PTSD, which frequently co-occurs with PAE and which may have detrimental effects on young children’s neurodevelopmental outcomes.

## Aim of the study

This study aimed to investigate the association of PAE and early neurodevelopment through 2 years of age, adjusting for socio-demographic and psychosocial factors in the Drakenstein Child Health Study (DCHS), a South African birth cohort study.

## Materials and methods

### Design and setting

This study formed part of the DCHS, a multidisciplinary birth cohort study investigating the early determinants of child health (Stein *et al.*, 2015; Zar *et al.*, 2016; Donald *et al.*, 2018). The DCHS enrolled pregnant women (20–28 weeks’ gestation) from two primary health care clinics, Mbekweni (a predominantly black African community) and TC Newman (a mixed-ancestry community) in the Western Cape, South Africa. Both communities are characterised by low socio-economic status (SES) and a high prevalence of multiple psychosocial risk factors (Zar *et al.*, 2016). Pregnant women were eligible to participate if they were 18 years or older, had access to one of the two primary health care clinics for antenatal care and had stated no intention to move out of the district within the following year. Mother–child dyads were followed longitudinally until children were at least 6 years of age.

### Participants

This study utilised a subgroup from the DCHS. The PAE group comprised mothers with a minimum score of 11 on the alcohol questions of the Alcohol, Smoking and Substance Involvement Screening Test (ASSIST; Humeniuk *et al.*, 2008). A follow-up cohort completed a measure on alcohol questions at 3–6 weeks and 24 months of age. Mothers were asked postpartum to provide a positive history of alcohol use in any of the three trimesters of pregnancy at levels consistent with the World Health Organization’s moderate-to-severe alcohol use. The unexposed group included children whose mothers had a score less than 11 on the ASSIST antenatally. After birth, infants identified were included for the study unless the mothers had a positive urine screen for any other drug abuse (opiates, marijuana, cocaine, methamphetamine and barbiturates). Infants born prematurely or with any other congenital malformations as well as sets of twins and triplets were excluded from the study.

**Fig. 1.**
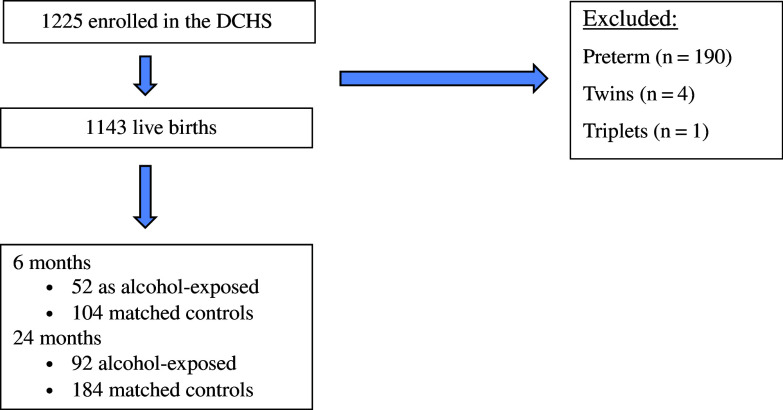
Study sample selection.

In total, there were 1143 live births in the DCHS (see Donald *et al.*, 2018). A subsample of the larger cohort completed the Bayley Scales of Infant Development-III (BSID-III) at 6 months (*n* = 260), whereas the full cohort was invited to participate at 24 months (*n* = 734) making a larger sample available. At 6 and 24 months of age, a subset of infants and toddlers were selected whose mothers reported moderate-to-severe levels of alcohol consumption and for whom BSID-III data were available. Of the 260 infants, 52 were exposed to alcohol at 6 months, and of the 734 toddlers, 92 were exposed to alcohol at 24 months. The unexposed group comprised 104 at 6 months and 184 at 24 months. Unexposed control children were randomly matched for maternal education and clinic site in a 1:2 ratio (Fig. 1).

### Measures

Participants were asked to complete self-reported and clinician-administered measures at antenatal and postnatal study visits in their preferred language, English, Afrikaans or isiXhosa. At the point of assessments (6 and 24 months of age), every effort was made to ensure a safe, anonymous, confidential and supportive environment. Translation of the measures from English to Afrikaans and isiXhosa included a standard forward and back-translation process (see Stein *et al.*, 2015). Prior to the administration of the measures, adult mothers or legal guardians of the children received enough information about the study and were asked to complete an informed consent form in their preferred language.

Maternal socio-demographic, psychosocial and infant measures for this study have previously been described (Stein *et al.*, 2015; Donald *et al.*, 2018) and included:

#### Socio-demographic measures

Measures included data on SES (maternal income, education, employment status and asset sum), marital status and HIV status (Myer *et al.*, 2008). Higher scores on this validated composite score indicated higher SES.

#### Psychosocial measures

Measures included data on composite scores of maternal smoking (cigarette and cannabis use) and psychological variables (PTSD and depression) administered antenatally. Maternal smoking was assessed using the ASSIST (Humeniuk *et al.*, 2008), maternal PTSD was assessed using the Modified Post-traumatic Stress Disorder Symptom Scale (Foa *et al.*, 1993) and maternal depression was assessed using the Beck Depression Inventory (Beck *et al.*, 1996).

Composite scores were created for maternal smoking and psychological variables. The indicators for SES included maternal income, education, employment status and asset sum; smoking included cigarette and cannabis use and psychological variables included PTSD and depression. Composite variables were used to combine data into a single score as they are considered more robust than a unidimensional measure (Field, 2013).

#### ASSIST

As above, the ASSIST assessed alcohol or substance use. This measure includes seven items with scores from 0 to 10 for alcohol and 0 to 3 for illicit drugs indicating low risk, 11 to 26 for alcohol and 4 to 26 for illicit drugs indicating moderate risk, and above 26 as high risk of severe problems, with the likelihood of alcohol dependence (Group, 2002). The higher the score, the greater the alcohol-related risk. The ASSIST has good reliability and validity in several countries including Australia, Brazil, Ireland, India, Israel, the Palestinian Territories, Puerto Rico, the United Kingdom and Zimbabwe (Group, 2002) and in South Africa (Humeniuk *et al.*, 2008).

#### Bayley Scales of Infant Development-III

The BSID-III was conducted at the 6- and 24-month visits, to assess child development in infants and toddlers between 0 and 42 months (Bayley, 2006). This is an international, well-validated test that was used to measure language and motor development. The BSID-III has been standardised with a stratified sample of 1000 children ranging from 0 to 42 months that was representative of the US population with respect to gender, race/ethnicity, geographic region and parent education level having high reliability and validity (Bayley, 2006). The Bayley-III has been shown to be a reliable tool for use among the South African population (Rademeyer & Jacklin, 2013).

The motor scale evaluated early fine and gross motor development (Bayley, 2006). The gross motor subset included 72 items that assessed movement of the limbs, static positioning (e.g. sitting and standing) and dynamic movement, including locomotion, coordination, balance and motor planning. The fine motor subtest included 66 items that assessed prehension, perceptual-motor integration, motor planning, speed, visual tracking, reaching, object grasping, object manipulation, functional hand skills and responses to tactile information. The motor assessments were administered using directly observed items for the infant and toddler (Bayley, 2006).

The language scale assessed receptive and expressive communication and was directly administered to the infant or toddler (Bayley, 2006). The receptive communication subtest includes 49 items that assessed pre-verbal behaviour, vocabulary development (identifying objects and pictures), understanding morphological development (pronouns and prepositions), morphological markers (e.g. plural, tense markings and the possessive), social referencing and verbal comprehension (Bayley, 2006). The expressive communication subtest included 48 items that assessed pre-verbal communication (babbling and gesturing), vocabulary development (naming objects, pictures or naming attributes) and morpho-syntactic development. Composite scores were based on the composite equivalents of the scaled scores. Scaled scores were based on scores with a mean of 10 and a standard deviation of 3 and range from 1 to 19. At 6 months, scaled scores were corrected for prematurity. The assessors were trained by a paediatric neurologist who ensured quality control and scoring precision. A trained paediatric occupational therapist or physiotherapist administered the BSID-III scales in the home language of the infants and toddlers. The assessors had background experience in paediatric clinical and research environments and were blinded to the exposure status of the children.

The DCHS was approved by the Faculty of Health Sciences Human Research Ethics Committees of the University of Cape Town and Stellenbosch University in South Africa, and by the Western Cape Department of Health Provincial Research Committee. All study participants provided written informed consent.

### Statistical analysis

The data were analysed using descriptive statistics which included frequencies and percentages for categorical data, while means (SD) were presented for normally distributed data. Medians [interquartile range (IQR)] were presented for data that were not normally distributed and for all BSID-III scores. For comparisons between alcohol-exposed and -unexposed children, chi-squared tests were used for categorical variables, while *t*-tests or, in the case of data that were not normally distributed, Mann–Whitney *U*-test was used. Variables that were associated with PAE at an alpha level of 0.05 or less were included in the final model to determine whether the outcome measures that were significantly associated with PAE remained significant after adjusting for potential confounders (see Appendices A and B). Multiple hierarchical regression was used to explore the associations of PAE with motor and language development. The model was adjusted for the maternal socio-demographic and psychosocial confounding variables, which are known to be associated with child neurodevelopment (motor, language and cognitive outcomes). Potential confounding variables included composites of SES (Fried & Watkinson, 1988), smoking (cigarette and cannabis) and psychological variables (Fried & Watkinson, 1990), and child’s body mass index (BMI) z-score and child’s nutritional status according to their gender and age. Significance was set at 0.05, and 95% confidence intervals (CIs) were reported for all estimates, where applicable.

## Results

The maternal and child socio-demographic and psychosocial characteristics are presented in Table 1. At 6 months, the median maternal age at enrolment was 24 years (IQR = 21–30). In the alcohol group, 15.4% of the mothers were HIV infected, 46.2% were classified as having PTSD and depression and in the unexposed group, 14.4% of the mothers were HIV infected and 55.8% had PTSD and depression. At 24 months, the median maternal age at enrolment was 26 years (IQR = 22–31). In the alcohol-exposed group, 16.3% of the mothers were HIV infected and 44.6% were classified as having PTSD and depression and in the unexposed group, 16.8 % of the mothers were HIV infected and 43.1% had PTSD and depression. There were no differences across the groups in socio-demographic or psychosocial variables, except for smoking, where mothers who consumed alcohol were more likely to smoke at both 6 (65.4% vs. 37.5%, respectively, *p* = 0.001) and 24 months of age (69.6% vs. 37.5%, respectively, *p* = 0.001). There were no significant differences between the exposed and unexposed groups regarding infants’ birth weight and BMI.

**Table 1.** Maternal and infant baseline socio-demographic and psychosocial characteristics

	6 months			24 months		
	Alcohol exposed	Unexposed	*p*-Value	Alcohol exposed	Unexposed	*p*-Value
Maternal variables	52 (33.3)	104 (66.7)		92 (33.3)	184 (66.7)	
Age, *n* (%)						
18–29 years	36 (69.2)	79 (76.0)	0.185	59 (64.1)	126 (68.5)	0.837
30–39 years	15 (28.8)	22 (24.0)		30 (32.6)	51 (27.7)	
40–49 years	1 (1.9)	0 (0)		3 (3.3)	1 (3.8)	
Study site, *n* (%)						
Mbkweni	19 (36.5)	38 (36.5)	1.000	32 (34.8)	66 (35.9)	0.484
TC Newman	33 (63.5)	66 (63.5)		60 (65.2)	118 (64.1)	
SES, *n* (%)						
Low levels of SES	16 (31.4)	34 (33.3)	0.448	29 (31.9)	65 (35.7)	0.601
Low–medium level SES	14 (27.5)	31 (30.4)		29 (31.9)	54 (29.7)	
Medium-to-high level of SES	16 (31.4)	21 (20.6)		22 (24.2)	34 (18.7)	
High SES	5 (9.8)	16 (15.7)		11 (12.1)	29 (15.9)	
Education, *n* (%)						
Primary	4 (7.7)	7(6.7)	0.952	13 (14.1)	24 (13.0)	0.968
Secondary	61 (92.3)	97 (93.3)		79 (85.9)	160 (87.0)	
Tertiary	0 (0)	0 (0)		0 (0)	0 (0)	
Marital status, *n* (%)						
Married or cohabiting	22 (42.3)	33 (31.7)	0.192	54 (58.7)	107 (58.2)	0.518
Other	30 (57.7)	71 (68.3)		38 (41.3)	77 (41.8)	
HIV status, *n* (%)						
Uninfected	44 (84.6)	89 (85.6)	0.873	77 (83.7)	153 (83.2)	0.528
Infected	8 (15.4)	15 (14.4)		15 (16.3)	31 (16.8)	
Smoking (cigarette and cannibas use), *n* (%)						
No	18 (34.6)	65 (62.5)	0.001*	28 (30.4)	115 (62.5)	0.001**
Yes	34 (65.4)	39 (37.5)		64 (69.6)	69 (37.5)	
Psychological variables (PTSD and depression), *n* (%)						
Absent	28 (53.8)	46 (44.2)	0.257	51 (55.4)	103 (56.9)	0.458
Present	24 (46.2)	58 (55.8)		41 (44.6)	78 (43.1)	
Child variables						
Sex, *n* (%)						
Male	26 (50.0)	60 (57.7)	0.362	50 (54.3)	102 (55.4)	0.482
Female	26 (50.0)	44 (42.3)		42 (45.7)	82 (44.6)	
Birth weight, *n* (%)						
>1500	1 (1.9)	0 (0.0)	0.405	3 (3.3)	2 (1.1)	0.089
1500 > 2500	8 (15.4)	11 (10.6)		14 (15.2)	29 (15.8)	
2500 > 3500	36 (69.2)	76 (73.1)		66 (71.7)	117 (63.6)	
<3500	7 (13.5)	17 (16.3)		9 (9.8)	36 (19.6)	
BMI z-score, median (IQR)	−0.005 (−0.75 to 0.77)	0.22 (−0.53 to 3.30)	0.118	0.18 (−0.48 to 0.97)	0.50 (−0.42 to 1.38)	0.113
Maternal age, years						
Median (IQR)	25 (21–31)	24 (21–29)	0.326	26 (22–31)	26 (22–31)	0.594
Gestational age, weeks						
Median (IQR)	39 (37–39)	39 (38–40)	0.138	39 (37–40)	39 (37–40)	0.811

* *p* < 0.05.

** *p* < 0.01.

Table 2 compares the median scores of the alcohol-exposed and -unexposed groups for motor and language development at both 6 and 24 months of age. At 6 months, alcohol-exposed infants had significantly lower median scores for gross and fine motor functioning compared with the unexposed infants [gross: median scores = 9.0 (IQR = 7.2–11.0) vs. 11.0 (IQR = 9.0–12.0), respectively, *p* = 0.006; fine: median scores = 11.5 (IQR = 10.0–13.0) vs. 13.0 (IQR = 12.0–15.0), respectively, *p* = 0.001)]. At 24 months, there were no significant differences, although there remained a trend towards impairment for fine motor functioning in exposed children [median scores = 8.0 (IQR = 7.0–11.0) vs. 9.0 (IQR = 8.0–11.0), respectively, *p* = 0.068]. There were no significant differences in language and cognitive functioning at 6 or at 24 months.

**Table 2.** Motor, language and cognitive development in the exposed and unexposed groups at 6 and 24 months of age

BSID-III subdomains	Alcohol-exposed median (IQR)	Unexposed median (IQR)	95% CI	*p*-Value	Alcohol-exposed median (IQR)	Unexposed median (IQR)	95% CI	*p*-Value
	*N* = 52	*N* = 104			*N* = 92	*N* = 184		
Gross motor	9.0 (7.2–11.0)	11.0 (9.0–12.0)	0.003–0.006	0.006**	8.0 (7.0–9.8)	9.0 (7.0–10.0)	0.20–0.19	0.196
Fine motor	11.5 (10.0–13.0)	13.0 (12.0–15.0)	0.001–0.001	0.001**	8.0 (7.0 –11.0)	9.0 (8.0–11.0)	0.06–0.07	0.068
Receptive communication	9.0 (8.0–11.0)	10.0 (8.3–12.0)	0.60–0.62	0.608	7.0 (5.0–8.0)	7.0 (6.0–9.0)	0.85–0.84	0.843
Expressive communication	10.0 (7.0–13.0)	10.0 (8.0–13.0)	0.99–0.99	0.991	7.0 (6.0–9.0)	7.0 (6.0–9.0)	0.74–0.75	0.743
Cognitive functioning	9.0 (7.0–11.0)	10.0 (8.0–11.0)	0.23–0.24	0.239	7.0 (6.0–8.0)	8.0 (6.0–8.0)	0.52–0.51	0.518

* *p* < 0.05.

** *p* < 0.01.

Table 3 demonstrates the regression analysis for gross and fine motor functioning. PAE was significantly associated with gross motor functioning [odds ratio (OR) = 0.16, 95% CI = 0.06–0.44, *p* = 0.001] and fine motor functioning (OR = 0.16, 95% CI = 0.06–0.46, *p* = 0.001) after controlling for BMI, SES, and smoking and psychological variables at 6 months of age. BMI was significantly associated with both gross (OR = 0.83, 95% CI = 0.57–1.21, *p* = 0.001) and fine motor functioning (OR = 0.67, 95% CI = 0.46–0.97, *p* = 0.004), while SES was significantly associated with gross motor functioning (OR = 2.28, 95% = CI 1.24–4.19, *p* = 0.001) at 6 months.

**Table 3.** Coefficients for predictors in final model of gross motor functioning at 6 months of age (after adjusting for SES, smoking, PTSD and depression)

Variables	Gross motor functioning			Fine motor functioning		
	Odds ratio	95% CI	*p*-Value	Odds ratio	95% CI	*p*-Value
BMI	0.83	0.57–1.21	0.001**	0.67	0.46–0.97	0.035*
SES	2.28	1.24–4.19	0.009**	0.99	0.54–1.80	0.971
Smoking	1.35	0.49–3.74	0.566	0.69	0.25–1.88	0.473
Psychological variables	0.71	0.27–1.87	0.493	0.48	0.18–1.25	0.134
PAE	0.16	0.06–0.44	0.001**	0.16	0.06–0.46	0.001**

* *p* < 0.05.

** *p* < 0.01.

The final model explained a significant amount of variance in gross motor functioning (*F*(4) = 3.98, *p* = 0.002, adj *R* ^2^ = 0.10), and the *R* ^2^ showed that the amount of variance increased from 5% (BMI, SES, smoking, PTSD and depression) to 14% after adding PAE into the model (Appendix A). Similarly, the final model explained a significant amount of variation in fine motor functioning (*F*(4) = 3.66, *p* = 0.004, adj *R* ^2^ = 0.13), and the *R* ^2^ showed that the explained variance accounted for an extra 9% (0.04–0.13) in fine motor functioning (Appendix A).

## Discussion

This study comprehensively assessed motor, language and cognitive functioning in a population-based cohort over the first 2 years of life. The findings of our study indicated that PAE is associated with both gross and fine motor functioning at 6 months of age, even after adjusting for maternal socio-demographic and psychosocial factors. While PAE was not associated with receptive and expressive communication nor cognitive performance at either time point in this group, there remained a trend towards significance for poorer fine motor functioning at 24 months of age.

Our findings demonstrate that PAE is associated with deficits in motor functioning across the first 2 years of life. This is consistent with previously reported cohort studies in preschool age (Fried & Watkinson, 1988; Jacobson & Jacobson, 2002; Davies *et al.*, 2011). In particular, Fried and Watkinson (1988) found a significant association between PAE and motor functioning in early infancy (12 months), even after adjusting for maternal age, gestation, birth weight and parity but found the effect to wane at later ages. These same investigators continued to report a lack of association between PAE and motor outcomes in a follow-up of these children into school age but reported associations between PAE and language comprehension at 36 months (Fried & Watkinson, 1990). Important differences between our study and the cohort in these studies include middle-to-high-income samples, no control groups and the authors adjusted for primarily physical confounders (maternal age, gestation, birth weight and parity), but not psychosocial factors. Our study adds to the growing body of scientific evidence implicating PAE in motor functioning impairment at 6 months of age even after adjusting for important psychosocial factors such as PTSD and depression when compared to a matched control group.

In our cohort, PAE was not found to be associated with receptive or expressive communication or cognitive functioning at the age of 24 months. Previous studies have reported impairments in language and cognitive functioning in toddlers between the ages of 12 and 24 months (Fried & Watkinson, 1988; Davies *et al.*, 2011); however, reports indicated that as children grew into the school years, PAE was not significantly associated with language or cognitive outcomes (Coggins *et al.*, 2007) using standard measures. Lack of associated impact of PAE on early language outcomes in this study may, in part, be a result of language impairments being subtle in infancy, and it is therefore being more difficult to identify these outcomes than in other domains. It may be useful for future studies to consider the extent to which specific language outcomes affect the pragmatic or conversational patterns of children affected by PAE (not just general categories of receptive or expressive communication).

Additional limitations deserve consideration. Firstly, the substudy comprised a small sample size which may have limited the power to detect differences between the groups. Secondly, despite assurances of confidentiality, some women may have chosen not to disclose or minimise reporting alcohol use to the research teams and the low reported alcohol consumption may therefore represent an element of response bias. Thirdly, the BSID-III tool measures general ability in completing a given task but may have low sensitivity for detecting minor developmental impairments especially during infancy. Further, although this tool has been validated for use in South Africa, this study may not be generalisable to other populations.

A large proportion of very young children in LMICs do not reach their developmental potential due to a wide variety of socio-demographic and psychosocial factors that may impact early developmental outcomes. Our study, reporting the association of PAE and early motor functioning, is one of only a few studies that have additionally addressed important potential psychosocial confounders which frequently co-occur with alcohol use in these communities. These findings highlight the importance of identifying high-risk families in order to provide preventive interventions, particularly in antenatal clinics and early intervention services.

## Appendix A. Summary of regression analysis: regressing gross motor functioning onto BMI, SES, smoking, psychological variables risk and PAE at 6 months of age

**Table:**

Model	*R*	*R* ^2^	Adjusted *R* ^2^	*F*	*p*-Value
1	0.03	0.001	0.04	1.55	0.695
2	0.23	0.05	0.34	3.45	0.035
3	0.23	0.05	0.03	230	0.80
4	0.23	0.05	0.02	1.75	0.14
5	0.37	0.14	0.100	3.98	0.002**

Predictors: Model 1 – BMI; Model 2 – BMI and SES; Model 3 – BMI, SES and smoking; Model 4 – BMI, SES, smoking and psychological variables; Model 5 – BMI, SES, smoking, psychological variables and PAE.

* *p* < 0.05.

** *p* < 0.01.

## Appendix B. Summary of regression analysis: regressing fine motor functioning onto SES, smoking, psychological variables risk and PAE at 6 months of age

**Table:**

Model	*R*	*R* ^2^	Adjusted *R* ^2^	*F*	*p*-Value
1	0.07	0.005	−0.001	0.79	0.375
2	0.12	0.01	0.001	0.99	0.371
3	0.18	0.03	0.01	1.46	0.229
4	0.21	0.04	0.01	1.44	0.225
5	0.36	0.13	0.09	3.66	0.004**

Predictors: Model 1 – BMI; Model 2 – BMI and SES; Model 3 – BMI, SES and smoking; Model 4 – BMI, SES, smoking, SES and psychological variables; Model 5 – BMI, SES, smoking, SES, psychological variables and PAE.

* *p* < 0.05.

** *p* < 0.01.

## References

[ref1] Adnams CM , Kodituwakku PW , Hay A , Molteno CD , Viljoen D and May PA (2001) Patterns of cognitive-motor development in children with fetal alcohol syndrome from a community in South Africa. Alcoholism: Clinical and Experimental Research 25, 557–562. doi: 10.1111/j.1530-0277.2001.tb02250.x 11329496

[ref2] Bayley N (2006) Bayley Scales of Infant and Toddler Development. San Antonio, TX: Pearson.

[ref3] Beck AT , Steer RA and Brown GK (1996) Beck depression inventory-II. San Antonio 78, 490–498.

[ref4] Coggins TE , Timler GR and Olswang LB (2007) A state of double jeopardy: impact of prenatal alcohol exposure and adverse environments on the social communicative abilities of school-age children with fetal alcohol spectrum disorder. Language, Speech, and Hearing Services in Schools 38, 117–127. doi: 10.1044/0161-1461(2007/012) 17428958

[ref5] Comasco E , Rangmar J , Eriksson UJ and Oreland L (2018) Neurological and neuropsychological effects of low and moderate prenatal alcohol exposure. Acta Physiologica 222, 1–18. doi: 10.1111/apha.12892 28470828

[ref6] Cone-Wesson B (2005) Prenatal alcohol and cocaine exposure: influences on cognition, speech, language, and hearing. Journal of Communication Disorders 38, 279–302. doi: 10.1016/j.jcomdis.2005.02.004 15862811

[ref7] Davies L , Dunn M , Chersich M , Urban M , Chetty C , Olivier L and Viljoen D (2011) Developmental delay of infants and young children with and without fetal alcohol spectrum disorder in the Northern Cape Province, South Africa. African Journal of Psychiatry 14, 298–305. doi: 10.4314/ajpsy.v14i4.7 22038428

[ref8] Donald KA , Hoogenhout M , du Plooy CP , Wedderburn CJ , Nhapi RT , Barnett W , Hoffman N , Malcolm-Smith S , Zar HJ and Stein DJ (2018) Drakenstein Child Health Study (DCHS): investigating determinants of early child development and cognition. BMJ Paediatrics 2, 1. doi: 10.1136/bmjpo-2018-000282 PMC6014194 29942867

[ref9] Group WAW (2002) The alcohol, smoking and substance involvement screening test (ASSIST): development, reliability and feasibility. Addiction 97, 1183–1194. doi: 10.1046/j.1360-0443.2002.00185.x 12199834

[ref10] Field A (2013) Discovering Statistics using IBM SPSS Statistics. North America: Sage, pp. 1–915.

[ref11] Flak AL , Su S , Bertrand J , Denny CH , Kesmodel US and Cogswell ME (2014) The association of mild, moderate, and binge prenatal alcohol exposure and child neuropsychological outcomes: a meta-analysis. Alcoholism: Clinical and Experimental Research 38, 214–226. doi: 10.1111/acer.12214 23905882

[ref12] Foa EB , Riggs DS , Dancu CV and Rothbaum BO (1993) Reliability and validity of a brief instrument for assessing post-traumatic stress disorder. Journal of Traumatic Stress 6, 459–473. doi: 10.1002/jts.2490060405

[ref13] Fried P and Watkinson B (1988) 12- and 24-Month neurobehavioural follow-up of children prenatally exposed to marihuana, cigarettes and alcohol. Neurotoxicology and Teratology 10, 305–313. doi: 10.1016/0892-0362(88)90032-3 3226373

[ref14] Fried PA , O’connell CM and Watkinson B (1992) 60- and 72-Month follow-up of children prenatally exposed to marijuana, cigarettes, and alcohol: cognitive and language assessment. Journal of Developmental and Behavioral Pediatrics 13, 299–311. doi: 10.1097/00004703-199212000-00001 1469105

[ref15] Fried PA and Watkinson B (1990) 36- and 48-Month neurobehavioral follow-up of children prenatally exposed to marijuana, cigarettes, and alcohol. Journal of Developmental and Behavioral Paediatrics 11, 49–58. doi: 10.1097/00004703-199004000-00003 2324288

[ref16] Henderson J , Gray R and Brocklehurst P (2007) Systematic review of effects of low–moderate prenatal alcohol exposure on pregnancy outcome. BJOG: An International Journal of Obstetrics & Gynaecology 114, 243–252. doi: 10.1111/j.1471-0528.2006.01163.x 17233797

[ref17] Hendricks G , Malcolm-Smith S , Adnams C , Stein DJ and Donald KA (2018) Effects of prenatal alcohol exposure on language, speech and communication outcomes: a review longitudinal studies. Acta Neuropsychiatrica 31, 74–83. doi: 10.1017/neu.2018.28 30449293 PMC7056946

[ref18] Humeniuk R , Ali R , Babor TF , Farrell M , Formigoni ML , Jittiwutikarn J , De Lacerda RB , Ling W , Marsden J , Monteiro M and Nhiwatiwa S (2008) Validation of the alcohol, smoking and substance involvement screening test (ASSIST). Addiction 103(6), 1039–1047. doi: 10.1111/j.1360-0443.2007.02114.x 18373724

[ref19] Hutchinson D , Youssef GJ , McCormack C , Wilson J , Allsop S , Najman J , Elliott E , Burns L , Jacobs S , Honan I and Rossen L (2019) Correction to: Prenatal alcohol exposure and infant gross motor development: a prospective cohort study. BMC Pediatrics 19(1), 222. 31088407 10.1186/s12887-019-1516-5 PMC6515673

[ref20] Jacobson JL and Jacobson SW (2002) Effects of prenatal alcohol exposure on child development. Alcohol Research and Health 26, 282–286. doi: 10.1111/j.1530-0277.1993.tb00744.x 12875038 PMC6676687

[ref21] Kalberg WO , Provost B , Tollison SJ , Tabachnick BG , Robinson LK , Eugene Hoyme H , Trujillo PM , Buckley D , Aragon AS and May PA (2006) Comparison of motor delays in young children with fetal alcohol syndrome to those with prenatal alcohol exposure and with no prenatal alcohol exposure. Alcoholism: Clinical and Experimental Research 30, 2037–2045. doi: 10.1111/j.1530-0277.2006.00250.x 17117969

[ref22] Kaplan-Estrin M , Jacobson SW and Jacobson JL (1999) Neurobehavioral effects of prenatal alcohol exposure at 26 months. Neurotoxicology and teratology 21(5), 503–511. 10492385 10.1016/s0892-0362(99)00031-8

[ref23] Keen CL , Uriu-Adams JY , Skalny A , Grabeklis A , Grabeklis S , Green K , Yevtushok L , Wertelecki WW and Chambers CD (2010) The plausibility of maternal nutritional status being a contributing factor to the risk for fetal alcohol spectrum disorders: the potential influence of zinc status as an example. Biofactors 36(2), 125–135. doi: 10.1002/biof.89 20333752 PMC2927848

[ref24] Kodituwakku P (2007) Defining the behavioral phenotype in children with fetal alcohol spectrum disorders: a review. Neuroscience & Biobehavioral Review 31, 192–201. doi: 10.1016/j.neubiorev.2006.06.020 16930704

[ref25] Kodituwakku PW , Segall JM and Beatty GK (2011) Cognitive and behavioral effects of prenatal alcohol exposure. Future Neurology 6, 237–259. doi: 10.2217/fnl.11.4

[ref26] Mattson SN , Crocker N and Nguyen TT (2011) Fetal alcohol spectrum disorders: neuropsychological and behavioral features. Neuropsychology Review 21, 81–101. doi: 10.1007/s11065-011-9167-9 21503685 PMC3410672

[ref27] May PA , Gossage JP , Marais AS , Hendricks LS , Snell CL , Tabachnick BG , Stellavato C , Buckley DG , Brooke LE and Viljoen DL (2008) Maternal risk factors for fetal alcohol syndrome and partial fetal alcohol syndrome in South Africa: a third study. Alcoholism: Clinical and Experimental Research 32(5), 738–753. 10.1111/j.1530-0277.2008.00634.x 18336634

[ref28] Myer L , Stein DJ , Grimsrud A , Seedat S and Williams DR (2008) Social determinants of psychological distress in a nationally-representative sample of South African adults. Social Science & Medicine 66, 1828–1840. doi: 10.1016/j.socscimed.2008.01.025 18299167 PMC3203636

[ref29] Nayak RB and Murthy P (2008) Fetal alcohol spectrum disorder. Indian Pediatrics 977, 977–983. 19129565

[ref30] O’Leary C , Zubrick SR , Taylor CL , Dixon G and Bower C (2009) Prenatal alcohol exposure and language delay in 2-year-old children: the importance of dose and timing on risk. Pediatrics 123, 547–554. 19171621 10.1542/peds.2008-0459

[ref31] O’Leary CM (2004) Fetal alcohol syndrome: diagnosis, epidemiology, and developmental outcomes, Journal of Paediatrics and Child Health 40, 2–7. doi: 10.1111/j.1440-1754.2004.00280 14717994

[ref32] Olivier L , Viljoen D and Curfs L (2016) Fetal alcohol spectrum disorders: prevalence rates in South Africa: the new millennium. South African Medical Journal 106(1), 103–106. doi: 10.7196/SAMJ.2016.v106i6.11009 27245541

[ref33] Popova S , Lange S , Probst C , Gmel G and Rehm J (2017) Estimation of national, regional, and global prevalence of alcohol use during pregnancy and fetal alcohol syndrome: a systematic review and meta-analysis. The Lancet Global Health 5(3), 290–299. doi: 10.1016/S2214-109X(17)30021-9 28089487

[ref34] Rademeyer V and Jacklin L (2013) A study to evaluate the performance of black South African urban infants on the Bayley Scales of Infant Development III. South African Journal of Child Health 7, 54–59. doi: 10.7196/sajch.547

[ref35] Safe B , Joosten A and Giglia R (2018) Assessing motor skills to inform a fetal alcohol spectrum disorder diagnosis focusing on persons older than 12 years: a systematic review of the literature. Journal of Population Therapeutics and Clinical Pharmacology 25, 25–38. doi: 10.22374/1710-6222.25.1.3 29949680

[ref36] Sokol RJ , Delaney-Black V and Nordstrom B (2003) Fetal alcohol spectrum disorder. JAMA 290, 2996–2999. doi: 10.1001/jama.290.22.2996 14665662

[ref37] Stein D , Koen N , Donald K , Adnams C , Koopowitz S , Lund C , Marais A , Myers B , Roos A and Sorsdahl K (2015) Investigating the psychosocial determinants of child health in Africa: the Drakenstein Child Health Study. Journal of Neuroscience Methods 252, 27–35. doi: 10.1016/j.jneumeth.2015.03.016 25797842 PMC4556362

[ref38] Viholainen H , Ahonen T , Lyytinen P , Cantell M , Tolvanen A and Lyytinen H (2006) Early motor development and later language and reading skills in children at risk of familial dyslexia. Developmental Medicine and Child Neurology 48(5), 367–373. doi: 10.1017/S001216220600079X 16608545

[ref39] Zar HJ , Barnett W , Stadler A , Gardner-Lubbe S , Myer L and Nicol MP (2016) Aetiology of childhood pneumonia in a well vaccinated South African birth cohort: a nested case-control study of the Drakenstein Child Health Study. The Lancet Respiratory Medicine 4(6), 463–472. doi: 10.1016/S2213-2600(16)00096-5 27117547 PMC4989125

